# Decorin Core Protein (Decoron) Shape Complements Collagen Fibril Surface Structure and Mediates Its Binding

**DOI:** 10.1371/journal.pone.0007028

**Published:** 2009-09-15

**Authors:** Joseph P. R. O. Orgel, Aya Eid, Olga Antipova, Jordi Bella, John E. Scott

**Affiliations:** 1 BioCAT and µCoSM Centres: Pritzker Institute of Biomedical Science and Engineering, Illinois Institute of Technology, Chicago, Illinois, United States of America; 2 CSRRI and Department of Biological, Chemical and Physical Sciences, Illinois Institute of Technology, Chicago, Illinois, United States of America; 3 Wellcome Trust Centre for Cell-Matrix Research, Faculty of Life Sciences, University of Manchester, Manchester, United Kingdom; 4 Chemical Morphology, Manchester University Medical School, Manchester University, Manchester, United Kingdom; Johns Hopkins School of Medicine, United States of America

## Abstract

Decorin is the archetypal small leucine rich repeat proteoglycan of the vertebrate extracellular matrix (ECM). With its glycosaminoglycuronan chain, it is responsible for stabilizing inter-fibrillar organization. Type I collagen is the predominant member of the fibrillar collagen family, fulfilling both organizational and structural roles in animal ECMs. In this study, interactions between decoron (the decorin core protein) and binding sites in the d and e_1_ bands of the type I collagen fibril were investigated through molecular modeling of their respective X-ray diffraction structures. Previously, it was proposed that a model-based, highly curved concave decoron interacts with a single collagen molecule, which would form extensive van der Waals contacts and give rise to strong non-specific binding. However, the large well-ordered aggregate that is the collagen fibril places significant restraints on modes of ligand binding and necessitates multi-collagen molecular contacts. We present here a relatively high-resolution model of the decoron-fibril collagen complex. We find that the respective crystal structures complement each other well, although it is the monomeric form of decoron that shows the most appropriate shape complementarity with the fibril surface and favorable calculated energies of interaction. One molecule of decoron interacts with four to six collagen molecules, and the binding specificity relies on a large number of hydrogen bonds and electrostatic interactions, primarily with the collagen motifs KXGDRGE and AKGDRGE (d and e_1_ bands). This work helps us to understand collagen-decorin interactions and the molecular architecture of the fibrillar ECM in health and disease.

## Introduction

Shape is arguably one of the most important issues in biology. Permanent and reproducible but not necessarily rigid molecular-scale shapes provide the framework in which nervous, circulatory and digestive systems develop, and undergo changes in molecular pathology [1]. On the larger scale, animal shapes are maintained by their connective tissues, or more precisely by connective tissue extracellular matrices (ECMs). The shape and organization of each ECM depends on its collagen content and architecture, other ECM components, and cells being in the right place at the right time. Cells decide where they go, but maintaining their position is an extracellular process and, in an apparent paradox, the particular ECM arrangement helps cells decide what cell type they should be [2]–[4]. Collagen fibrils are the main architectural element in tissues such as cartilage, tendon, skin and bones, to which they impart mechanical and tensile strength as well as functioning as an organizational scaffold for the ECM. The mutual orientation and separation of these collagen fibrils is, in part, determined by proteoglycans (PGs) in the form of interfibrillar bridges [5], even in animals as distant from mammals as the echinoderms [6]. These bridges are soluble, but can be seen by staining with the electron dense marker Cupromeronic Blue that is visible in electron microscopy [5], [7].

**Figure 1:**
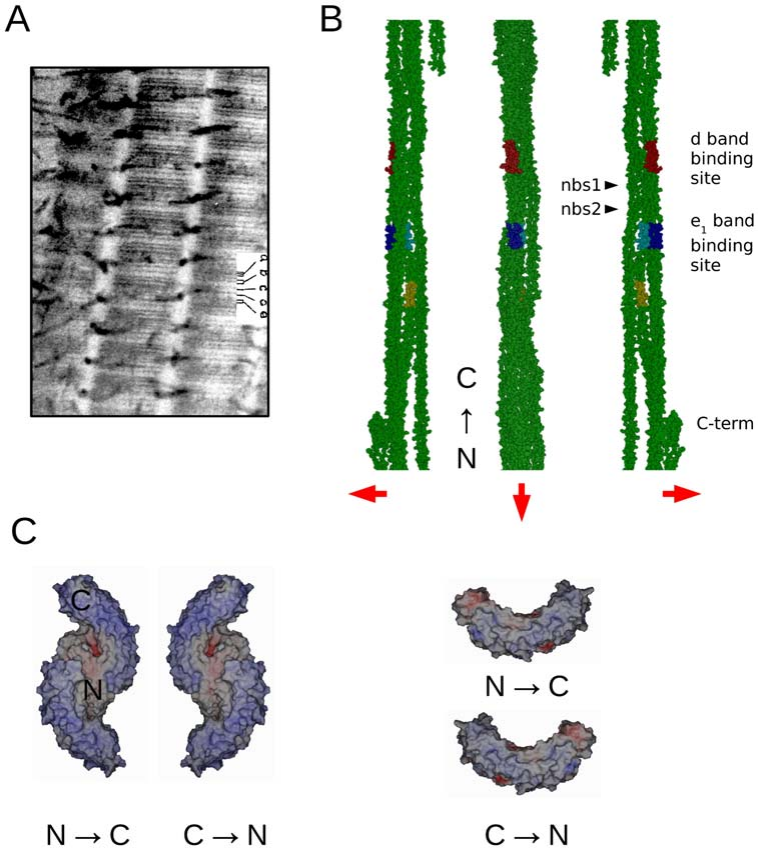
Consensus collagen sequences for decorin binding sites and decoron binding conformational orientations. A) Rabbit ventral skin, stained with Cupromeronic blue to demonstrate the proteoglycan filaments (which are about one D period apart) orthogonal to the collagen fibrils and subsequently with uranyl acetate to delineate the a–e banding pattern. B) The consensus decoron binding collagen sequences are shown here as coloured bands on a representation of the type I collagen microfibril [33], collagen N→C direction runs from bottom to top: Yellow = D∼2.65 (AOGDKGEAGPSG) *e*_2_-band site (partially accessible) Cyan = D∼2.74 (AOGDRGEOGPOG) *e*_1_-band site (partially accessible) Blue = D∼3.74 (AKGDRGETGPAG) *e*_1_-band site (fully accessible) Red = D∼3.87 (KNGDRGEOGPAG) *d*-band site (fully accessible). The positions of two non-binding sites used as negative controls in the molecular docking calculations are indicated as nbs1 and nbs2. The central representation is viewed from the exterior of the fibril surface, the left and right views are from within the fibril (red arrows point to fibril exterior). C) Electrostatic rendering of accessible surface area of a decoron in the Dec N→C or Dec C→N dimer (left) or monomer (right) conformations. For the Dec N→C conformation, the N-terminus is leftmost for the monomer, and in the central section of the dimer (inside of the dimer interface).

PG core proteins such as the small leucine rich repeat proteins (sLRRP) decoron, biglycan and fibromodulin bind to collagen fibrils at specific sites and carry anionic glycosaminoglycan (AGAG) strings which span the interfibrillar spaces [8]–[11] (Figure 1). These structures were called ‘shape modules’ [12] since they repeat regularly and help define ECM shapes. This means to at least an extent: we are held together by carbohydrate strings [13]. Thereby, although not at first obvious, we share a striking characteristic with plants. These carbohydrate strings are aggregated anti-parallel chains of dermatan [5], keratan and chondroitin sulphates (DS, KS & CS respectively), polymers that prefer a tape-like 2-fold helical configuration in H_2_O stabilised by hydrogen bonding and hydrophobic interactions [14]. Although these shape modules are elastic, AGAG-AGAG interactions break under stress but reform when the stress is removed as shown by *rheo* NMR [15], and/or they contain the elastic sugar L-iduronate (in DS). Direct proof of iduronate elasticity has been obtained by stretching individual AGAG molecules [16]. Iduronate-rich DS is characteristic of flexible tissues (skin and tendon), and DS chain lengths approximate the interfibrillar distances they span.

### Decorin

The elegant order shown in electron micrographs [5] later stimulated Ruoslahti to coin the term “decorin” for a DS-PG with a 40 kDa core protein that decorates the collagen fibrils [17]. The decorin-collagen fibril interaction is ubiquitous in every vertebrate ECM so far examined and is one of the most prominent in animal biology. This interaction must be highly specific, as electron micrographs obtained from a double-staining strategy show [5]. A heavy metal such as uranyl is used to produce the characteristic *a*–*e* banding pattern of collagen fibrils. This pattern, when matched against the Cupromeronic blue-stained PG, can be used to locate the PG molecules on the fibril surface [18]. Double staining shows that decorin most prominently occupies the *d* and *e*_1_ bands on type I collagen fibrils [19], (Figure 1). Immunoelectron microscopy results are also consistent with this localization [11], [20], [21]. Decorin and collagen, as reconstituted fibrils, isolated from the same tissue, rat tail tendon, interacted *in vitro* and decorin bound to the regions previously identified [22]. Further confirmation was obtained by synchrotron X-ray diffraction of Cupromeronic blue-stained tissue that had not been dehydrated or embedded [23].

Although the resolving power in these studies was not sufficient for the identification of individual amino acids, it was recognised that the *d* and *e* binding site regions contained elements of a common characteristic 11-amino acid sequence motif G*x*_1_ *x*_2_GDRGE*x*_3_GP [24], where *x*_1_ = K or A, *x*_2_ = N, K, S or P, and *x*_3_ = P or T, which was not present elsewhere in collagen types I-III. Possible complementary charge patterns on the amino acid sequences of several mammalian decorons were found; two motifs with opposite orientations: 243-RELH-246 on LRR repeat 10, and 101-KLER-104 on the LRR repeat 3–4 boundary [24], repeat numbering as in [25], where the uncharged, structural leucine residues would be equivalent spacers to the glycine residues in the collagen pattern.

Rotary-shadowed images of decorin preparations [24] revealed curved structures that were interpreted as individual molecules of decoron, with a “horseshoe” shape and an inner space that could accommodate one collagen molecule. At the time the only known crystal structure of an LRR protein was that of ribonuclease inhibitor which has a pronounced arched structure where a parallel β-sheet defines the concave side and the convex side is made of α-helices [26]. It was then hypothesized that the LRR structure of decoron would resemble that of ribonuclease inhibitor and that the internal cavity defined by the concave side would contain the binding site for one collagen molecule [27]. Several crystal structures of LRR molecules have since been determined, including decoron itself, where the degree of curvature is significantly less pronounced, see Bella, et al. 2008 [28] for a recent review on LRR structure. Thus, decoron falls into the more common category of LRR structures with a subtly curved shape as opposed to the pronounced arch shape of the ribonuclease inhibitor [28].

Lastly, the observed homodimerisation of decoron and the closely related PG biglycan, in solution and in crystal structures further complicates the understanding of decoron-collagen interaction [25], [29], [30]. The dimerization, which shows subnanomolar affinity [31], occurs through the concave sides of the LRR domains of these PGs and involves specific interactions. The concave side of LRR proteins contains the main binding sites for their ligands, with some exceptions [28]. Thus, dimerization of decoron and biglycan might seem incompatible with the expected mode of interaction of these PGs with collagen [32]. The recent determination of decoron and the *in situ* collagen fibril structures [4], [25], [33] open the possibility of exploring the postulated interaction of these two structures and their suggested interaction motifs described above.

## Results

We investigated the electrostatic landscape of the decoron-collagen fibril interaction under various modes of ligation, whilst taking into account spatial and steric considerations required of this complex. These included: the decoron monomer to collagen monomer and the decoron-dimer to collagen monomer and microfibril and fibrillar surface conformations in the two orientations (Dec N→C and Dec C→N, see methods and Figures 1–3) that the concave surface of the decoron molecule allows. We studied these in the context of the proposed decoron-binding sequence GAKGDRGETGP of the *e*_1_-microscopy band and the virtually identical GKNGDRGEPGP *d*-microscopy band sequence located approximately 0.13D (or 8.7 nm) apart within the gap region of the collagen fibril D-period (Figure 1).

**Figure 2 pone-0007028-g002:**
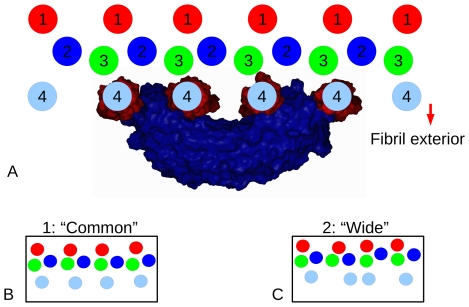
Molecular packing at the collagen fibril surface and decoron-fibril binding. A) Schematic, composite representation of a decoron molecule bound to the fibril surface (*e*_1_-band site) in the Dec N→C orientation; collagen monomers 1–4 from each microfibril are labelled. The four monomers in closest association with the docked decoron monomer are surface rendered in red (the decoron molecule is surface rendered in blue). B) Molecular packing structure of collagen monomers at the fibril surface at ∼0.74 D (*e*_1_-band site). 1: Represents the “common” arrangement of monomers around most of the fibril surface. 2: The maximum “wide” model.

**Figure 3 pone-0007028-g003:**
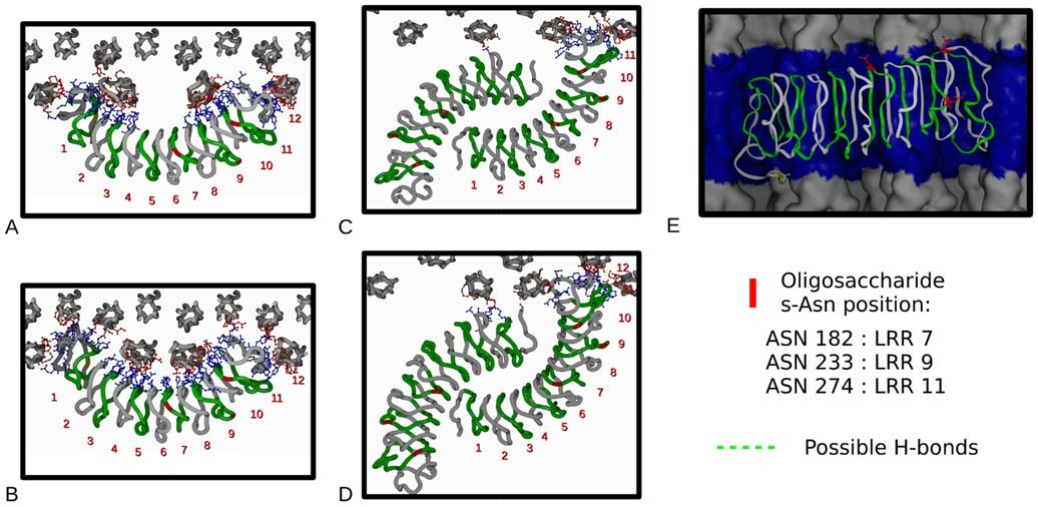
Decoron docking at the fibril surface for the common and wide arrangements at the *d* and *e_1_* binding sites. Side chains of the amino acid residues involved in the decoron/collagen interface are shown in red (collagen) or blue (decoron). Candidate interacting residues must be capable of forming at least one hydrogen bond, and to be less than 4 Å from a residue in the other molecule. Possible hydrogen bond interactions are shown in panels A–D for different model arrangements, all are in the Dec N→C orientation (see also Table S1). A) *e*_1_-band site, common conformation, decoron monomer. B) *e*_1_-band site, wide conformation, decoron monomer. C) *d*-band site, common conformation, decoron dimer. D) *d*-band site, wide conformation, decoron dimer. E) Decoron molecule docked at the common fibril surface model in the *e*_1_-band (blue). Oligosaccharide binding residues are shown, as is the AGAG chain binding N-terminal sequence (yellow). Note that the decoron molecule is tilted (∼8.5 degrees relative to the lateral plane of the collagen fibril) in its final energy minimized conformation, and the carbohydrate binding amino acid residues all appear to be fully accessible. In all A–E panels every other LRR is coloured green for reference (the first being LRR-1 at the N-term, then LRR-2 is gray, LRR-3 is green etc; the region N-terminal to LRR-1, including part of the capping structure, is shown in gray).

Forty-two decoron to collagen ‘receptor’ models based on the binding of the decoron concave surface to collagen were energy minimized as described in the methods. Their energies of association were estimated from the electrostatic-associative and desolvation-cost energies, after Camacho and Zhangs fastcontacts [34] definition and the number and location of specific interactions in the hydrogen bonding network of the ligand-receptor interface were calculated via the ‘whatif’ algorithm [35].

### Decoron crystal structure and fibrillar collagen packing structure are complementary

**Figure 4 pone-0007028-g004:**
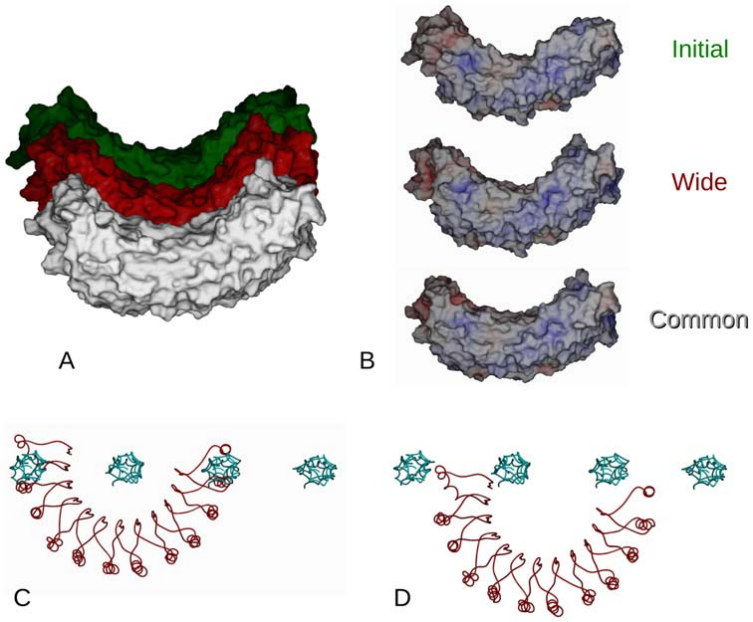
Curvature of the decoron monomer. A) Decoron's radius of curvature is only modestly changed upon binding fibril surface. The initial (crystal) structure and the wide and common conformation bound models of decoron were superimposed and then stacked along the molecules height axis to compare their overall shape and curvature B) Electrostatic rendering of decoron for each model referred to above. Note that for the common and wide bound models of decoron have more even, bracket shaped interior but are still not the highly arched shapes previously envisioned. C) In contrast to A and B, the decoron model is based on the ribonuclease inhibitor structure rather than the decoron crystal structure is highly curved. Here it is shown attempting to dock with the fibril surface as for Figures 2–3. Note the substantial molecular overlap that occurs when the decoron is docked to an individual collagen molecule, with the neighboring collagen molecules at the fibril surface. D) As C, except: the ribonuclease inhibitor based decoron molecule has been placed to avoid steric clashes, note that the receptor-ligand interface appears substantially less engaged than that seen in Figures 2–3.

**Figure 5:**
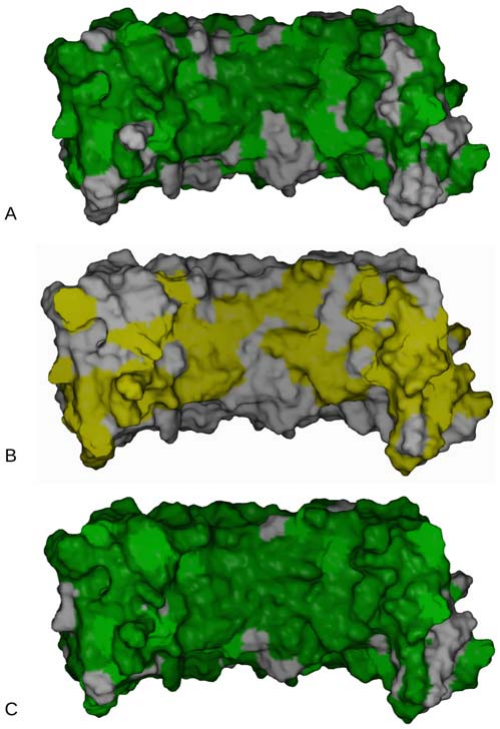
Homologous residues that contribute most to the energy of association. A) Homology between bovine decoron and bigylcan rendered as dark green for identical residues and light green for similar residues. Residues that are not common between the two proteins are rendered gray. B) Map of the decoron residues that most contribute to the energy of association with collagen for the 4 primary collagen receptors studied here (common and wide conformations for the d and e_1_ band sites), see Table S1 for reference. The darker shade of yellow corresponds to residues that are ranked as contributing highly to the ligand-receptor interaction (methods, Table S1). C) As for A), except comparison is between rat and bovine decoron sequences.

The most striking result is that the crystallographic structure of the decoron molecule [25] appears to have the right shape and dimensions for extensive interactions with the fibril surface [4], [36] in its monomeric form (Figures 2–3). Whilst a highly curved (as in ribonuclease inhibitor LRR structure) could not interact with the fibril surface without substantial steric overlap, the decoron molecular structure embraces multiple collagen monomers with only a very modest change to its radius of curvature (Figure 4). The predicted hydrogen bonding network between each decoron molecule and collagen is extensive (Figures 3, 5 and S1), sharing more than 30 decoron-collagen bonds in each of the eight decoron to collagen fibril surface models, although the relative free energies of these intermolecular interactions are not equivalent. While the candidate collagen receptor amino acid sequences are essentially the same between the *d* and *e*_1_-band binding sites, the specific conformations of the sites are not. Furthermore, the wide fibril surface packing conformations are dissimilar to the predominant, common, conformation in that the molecular packing of the collagen molecules presents significant differences in the placement of the collagen receptor sequences. Yet, decoron ligation for both the common and wide fibril conformations and at the *d* and *e*_1_ bands is likely to be strong.

**Figure 6:**
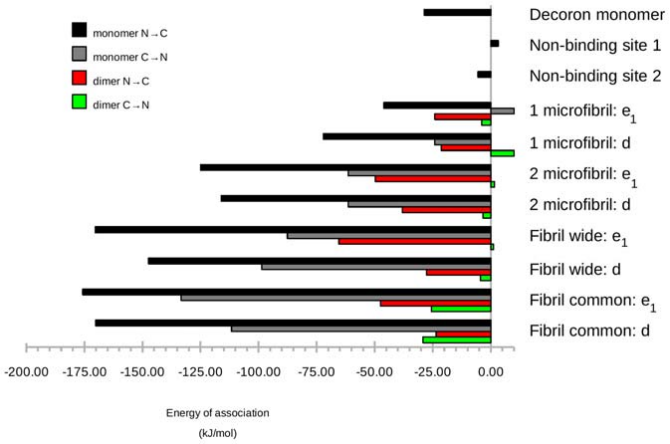
Free energy of collagen-ligand association for fibril surface receptor models.

It seems that rather than any small collection of amino acid interactions being specifically responsible for collagen-decoron ligation, there is an array of potential H-bonds (Figure S1 and Table S1). However, even the most favoured decoron model, the monomeric form, fails to interact strongly at the non-binding sites nbs1 and nbs2 (Figure 6). These non-binding sites are characterised by sparse opportunities for H-bonding or favourable polar interactions, as reflected by their poor energies of association (Figure 6).

### Most favourable models of decoron – collagen interactions

Figure 6 shows the relative energy of association for each of the ligand-receptor models. Whilst the most favourable mode of interaction with the fibril surface is that of the decoron monomer in the Dec N→C binding mode, several other interaction models are indicated to be at least moderately favourable. Interestingly, the calculated energy of association between two decorin monomers as present in the decoron crystal structure [25] is about –30 kJ/mol. Somewhat less favourable than the energies of association calculated for all the monomeric decoron to collagen receptor models, which range from –45 to –175 kJ/mol (with the exception of the non-binding site, negative control models, which show very small or positive energies of association). In contrast, the decoron dimer-to-receptor models showed association energies less than 50% of the best decoron monomer to equivalent receptor models (from –20 to –65 kJ/mol).

The ‘2-microfibril’ models presented receptor sites equivalent to the fibril surface common models, but restricted to the two microfibrils that directly interact with the concave face of decoron. Unsurprisingly, these showed a similar association pattern to the fibril surface models, although with the absence of the collagen molecules that contact the decoron terminal ends, the association energy is diminished (Figure 6). In the fibril surface models, most of the strongest electrostatic contacts occur between collagen and the N and C terminal arms in preference to the central LRR's of decoron in the common conformation (in the wide conformation it is the central LRR's that dominate the interaction). This point is emphasised for the common conformation *d*-band binding site, where the collagen monomers have much less contact with decorons LRR's 2 through 8 (the central section), than at the *e_1_*-band receptor site. In addition, monomer 3 at the *d*-band site, although less accessible to the fibril surface than monomer 4, makes a close approach to the tip of the decoron arms docked between the neighbouring monomer 4′s. Whereas at the *e_1_*-band, monomer 3 is further away, it and monomer 2 are close enough for at least one significant electrostatic contact (see Table S1, collagen R402C with decoron N30 and N37 make significant contributions to the energy of association).

### Decoron shape preserved; only modest changes upon binding the fibril surface

The decoron molecule shows the capacity to become strongly associated with more than one specific fibril surface ‘receptor’ (Figure 6). This does not come from any significant flexibility in the decoron conformation, which remained largely unchanged throughout the energy minimization (Figure 4); but from a redundancy of charged residues and potential H-bonding partners from the tips (concave *and* convex sides) of the N and C-terminal arms through to the interior of the concave surface. These terminal ends of the decoron stick into the fibril surface like a thumb and finger ‘pinching’ the concave interior collagen molecules.

Figure 4 shows a comparison of the decoron curvature from the initial crystal structure through to the final docked wide and common conformations. When superimposed, there is only a ∼2 degree shift in the curvature over the length of the most curved (common-bound) LRR structure. The N terminal end of the decoron molecule is not restricted by the conservative LRR structure and is more free to change conformation during energy minimization and therefore, presumably when docking with the collagen fibril receptor *in vivo*. Thus, the initial ‘banana’ shaped molecule adopts a (slightly) more evenly curved conformation when docked to the fibril surface. Nevertheless, the molecular shape is still far closer to the shallow curve observed in the relevant crystal structure [27] over and above that of the highly arched ribonuclease inhibitor based models (Figures 2–4, and S2).

### Decoron at the collagen fibril surface

At both the *d* and *e_1_* band sites, decoron binding is fundamentally the same; the charged concave surface of the monomer (Figures 1c and 2) faces the fibril surface, whilst charged residues at the N and C terminal ends penetrate the fibril surface into the solvent filled cleft (Figures 2 and 3). Here a substantial number of favourable electrostatic and H-bond partners are found across the complex interface (Figures 3, 5, and S1). In purely energetic terms, the common binding site with the decoron monomer is by far the most favourable interaction. The wide binding site should be considered to be the outlier variation in packing positions, whilst the common conformation is the average of the normal distribution in the molecular packing of the fibril [33]. Regardless of this, the association formed between the wide conformation and the decoron monomer is still significantly stronger than both the decoron-decoron dimer and the two non-binding site controls.

During the energy minimization of both the common and wide binding models, the decoron molecule shifted from its starting position that lay orthogonal to the fibril axis to eventually sit at an angle of ∼8.5 degrees (Figure 3e) relative to its starting position. This may be because it gave the best fit of the decoron arms into the spaces between the collagen monomers, but this hypothesis is less relevant for the wide fibril surface conformation which had no shortage of space in this regard. With this in mind, it is interesting to note a line of strong electrostatic interactions across the innermost core of the decoron concavity (Figure 5, between 4 and 13 degrees) that mirrors this tilt angle of attachment and this may explain the reproduction of this binding effect for the wide as well as common conformations (note also a similar line of homologous residues between bovine decoron and biglycan Figure 5a).

## Discussion

Two important questions decide the validity of the proposed models, viz. are the binding sites readily accessible and can they interact without mutual interference. Our results suggest the affirmative, with the liaison consisting of the decoron molecule concavity wrapped around two collagen molecules, with the tips of the decoron molecule penetrating the fibril surface. The convex (oligosaccharide-bearing) side remains exposed, with sugar residues completely free to interact (Figure 3e, see AGAG interacting N-terminus and Asn residues 182, 233, and 274).

### Decoron shape and putative binding

The banana shape derived from the X-ray data [25] is less curved than the ribonuclease inhibitor based model [27] that supposedly fits the rotary-shadowing data that show rounded, bracket-like to horseshoe shapes [24]. Our results indicate that fibril bound decoron has a shallow, rounded appearance (like that in the crystal structure) that maximises the interaction surface area (as is apparent in Figures 2 to [Fig pone-0007028-g003] [Fig pone-0007028-g004] 5). Our current work and previous X-ray data regarding the structure of decoron confirm the salient points from the rotary shadowed structure, in which the N-linked oligosaccharides are on the C-terminal branch of the convex side of the molecule. These are completely unrestricted when decoron is docked to the collagen fibril surface in the models presented here for the *d* and *e_1_*-band binding sites (Figures 2 and 3), as is the N-terminal attachment site for the AGAG chain (Ser 4, marked in yellow in Figure 3e).

Two putative collagen binding sites 104-RELK-101 and 243-RELH-246, respectively equidistant from the N and C terminals are located in LRR's 3–4 and 10, close to or within the concave face of the X-ray structure of decoron [24]. As Table S1 shows, RE of RELK and RE and H of RELH do contribute significantly to the energy of association. However, instead of a highly specific ‘perfect’ charge/no-charge complementarity, a more general redundancy in potential H-bond partners and favourable electrostatic surfaces appears to drive the association (Figures 5 and 6, and Table S1). However interference with the sequences in individual LRR's [37]–[40] could be expected to disturb decorons ability to bind collagen by disrupting the H-bonding network without necessarily being directly part of the intermolecular interaction.

Decoron presents in solution and crystals as a dimer, in which the putative binding sites on the concave face are buried, unavailable to interact with other ligands. The structural interactions proposed here require that the dimer disassociates to bind to collagen, a possibility already envisaged [29]. The postulated dissociation scenario is supported by the calculated energies of association of the collagen fibril-decoron complexes versus that of the decoron dimers (Figure 6). However, a pressing question is how does the same PG that appears relatively resistant to significant changes in curvature bind to such a wide range of fibril diameters: The curvature of the collagen fibril surface changes markedly from the very small (<20 nm) fibrils in developing tissue to the large >250 nm fibrils in aged tissue [7]. For larger fibril diameters, according to the model of Perumal, Antipova and Orgel [4], constructed of straight lattice sections forming a polygon rather than a round cylinder, based partly on observations of large, polygon shaped fibrils in rat tail tendon [41] and the model of Hulmes et al 1995 [42], the problem of decreased curvature is moot. The ‘common’ model presented here (after Perumal, Antipova and Orgel), has no curvature – it is planar. For smaller fibrils where the radius of curvature is relatively large, there is probably greater spacing between adjacent microfibrils at the fibril surface, which might resemble the ‘wide’ fibril model presented here [4]. Although providing a weaker attachment than the common model, it is still strong (Figure 6). The significant difference between the wide and common binding conformations is in *which* amino acid residues are involved in the interaction and not in how decoron curvature changes to accommodate a tightly curved small fibril. Again, decoron shows redundancy in this respect; although the involvement of LRRs 4 and 10 remains consistent in both models, their relative contributions of the strength of polar interactions is not equivalent (Table S1). The ligand-receptor interaction is facilitated by the establishment of a much broader hydrogen bonding network than previously envisioned – one that covers a large part of the concave surface and the terminal ‘thumb’ and ‘finger’ (N and C ends). Although the significance of collagen binding primarily with 2–3 decoron LRR's might appear diminished by these data, it is clear that the potential interaction between particular LRR's and collagen may be very strong (Figure 3 and Table S1).

### Decorins Elasticity is in its AGAG chain, strongly anchored by the protein core

Elasticity is fundamental to the functioning of ECMs, to provide reversible deformation during use. Recently it was pointed out that the interfibrillar AGAG bridges that attach to decoron must be elastic [13], suggesting that these bridges play some role in conveying elastic properties to interstitial tissues. Two mechanisms were demonstrated which provide reversible deformation, viz the presence of an elastic sugar unit (L-iduronate) in dermatan sulfate, proven directly by atomic force spectroscopy and a sliding filament mechanism, exemplified in hyaluronan by *rheo* NMR [15]. In partial relation to these facts, our decoron-collagen fibril structure is not affected by the relative orientation of collagen fibrils to each other, such as in the opposite polarities of fibrils observed in tendons for instance [5], [7], [10]. This is because the 180° ‘rotation’ implied in producing the opposite polarity does not alter the relationship with the glycan bridges, which locate to decoron by a xylosyl-serine single bond about which rotation is completely free in principle. Decoron's role is to supply a strong anchor for its elastic glycan bridge. Whilst decoron itself may be relatively inelastic in comparison, this would seem appropriate for the core protein whose primary structural role is to remain attached to the collagen fibril.

### Conclusion and summary

Decoron binds to the predominant conformation of the crystalline type I collagen fibril in monomeric rather than dimeric form, in a clearly preferred (Dec N→C) orientation. There is little difference in the binding affinity of the *d* and *e*_1_ band binding sites, although the specific conformation and packing of the collagen monomers produce differences in the strength of the interaction. Decoron for its part shows a robust redundancy in its mode of interaction, which appears to be mediated through substantial H-bonding contact between the large surface area in the concave face of the molecule and complementary H-bonding partner residues, particularly within the collagen binding sequences KXGDRGE (*d* band) and AKGDRGE (*e*_1_ band).

We hereby propose that each decoron monomer rather than dimer, binds to the collagen fibril surface and must interact with at least four separate collagen monomers (Figure 2 and S2 for a clearer view). The four collagen monomers are also members of 4 individual collagen microfibrils and decoron-collagen contacts seem to include monomer 3 at both the *d* and *e_1_* band sites, and monomer 2 at the *e_1_* band site. The docked arrangement naturally encompasses both the basic shape of the crystal structure of decoron and the fibril surface. Beyond the monomer needing to disassociate from the dimer, only modest changes to the decoron structure are required: side chain re-arrangement, small backbone shifts and a 2 degree (radius of curvature) shift from its starting position to make a gently curved, shallow bracket shape, which complements the shape of collagen fibril surface (Figures 1–3 and S2).

## Materials and Methods

The dimeric and monomeric forms of decoron (RCSB 1XCD) were docked in one of two orientations (Figure 1) as ‘ligands’ with the several collagen ‘receptor’ models at the *d* and *e*_1_ bands, plus two intermediate locations between the *d* and *e*_1_ bands. The receptor models included the fibril surface (convolution of RCSB 1Y0F along the ‘3.8’nm collagen packing lattice as described in Perumal, Antipova, Orgel 2008 [4]), a single microfibril, two side-by-side microfibrils (representing a minimal fibril surface model with only the decoron concave facing collagen molecules). The fibril surface models were further divided into the ‘common’ fibril conformation, or the ‘wide’ fibril conformation for both the *d* and *e*_1_ band sites (see Figures 2 and 3). Each model was energy minimized and its final energy of association (Figure 6) and H-bonding network were calculated (Figure S1 and Table S1) as below.

### Decoron model

The coordinates of two crystal structures of decoron, RCSB codes 1XCD and 1XKU were utilised for preliminary aspects of this study, and no significant differences were found between them in the context of the much lower resolution collagen microfibril/fibril structure. The dimeric form of decoron [25] as found within the crystal structure (RCSB coordinates) 1XCD was used for this study. For the monomeric form, the structural coordinates for decoron corresponding to 1XCD were modified by modelling the structure of the missing N and C-terminal amino acid residues (1–21, 327–330) of one of the two molecules found in the dimeric form to create one full length decoron molecule. The N-terminal sequence was modelled as a coil extending outwards of the molecule (towards the convex side), so that the AGAG binding sequence would be accessible outside of the solvent-filled cleft of the collagen fibril surface [4]. The very short addition to the C-terminal end was modelled as an extended conformation and both N and C-terminal additions were energy minimized as described below for the ligand-receptor modelling.

### Fibril surface binding sites for decoron

The structure of the fibril surface was constructed as described previously, by convolution of the coordinates for the collagen microfibril (RCSB 1Y0F) with the fibrillar packing lattice [4]. A model of four neighbouring microfibrils along the ‘3.8’ nm crystal lattice plane was generated after this fashion for each collagen binding site (*d* and *e*_1_ bands, plus two intermediate locations between the *d* and *e*_1_ bands). All coordinates 50 Å above and 50 Å below the plane of the decorin binding site/s were deleted for computational expediency whilst still allowing an excess of fibrillar context around the ∼25–40 Å of collagen-decoron interaction along the collagen molecular axis. Although highly ordered, the type I fibril surface packing conformation must occasionally deviate marginally from the ‘average’ crystallographic packing structure within each group of 4 microfibrils in both the lateral and axial planes, specifically:

1. ‘disorder’ determined variation in the lateral plane: There are thermal or glass-like state variations from the ‘ideal’ packing lattice in the order of +/− 0.31 nm in parts of the gap region [33], which were accounted for in the model of Perumal, Antiopva and Orgel 2008 [4]. This suggests that there may be regions on the surface of the fibril where the central two collagen monomers (molecules) dock with the concave surface of the decoron molecule are as much as 0.6 nm apart, but would not move more than 0.3 nm [33] closer to another collagen monomer from its ‘ideal’ position. We have modeled here just such an average maximum displacement packing model and termed it “wide” due to the increased distance between the collagen monomers neighboring the central two monomers in contact with decoron's concave surface, providing more space for docking decorons N and C terminal ‘arms’. The “common” conformation, which represents the intrinsically normalized crystal structure, likely represents the predominant state of packing at the fibril surface. However, we recognize that there is likely to be a continuum of packing arrangements between common and wide states, albeit highly biased towards the common conformation – or else the collagen fibril lattice could not be crystalline by definition.
2. non-disordered variation between *e*_1_ and *d* band binding sites (axial plane) is accounted for simply from the microfibrillar structure: The collagen monomers are *not* ‘straight-rods” in the gap region. Each adopts different molecular paths in the gap region as each molecular segment progress from 1 to 2, 2 to 3, 3 to 4 and 4 to 5 in the next D-period. The *e*_1_ and *d* band arrangements are similar, but non-identical despite the close sequence similarities: the actual placement of the collagen molecules in three dimensions is different between these two regions of the collagen molecules.

### Docking and Energy Minimization of Model Coordinates

The fibril surface conformation provided significant stringency regarding the mode of decoron binding, limiting the options to a choice between which way round the N-C direction of decorons concave side faces the fibril surface. Either the N to C directions runs left to right as viewed from the fibril exterior (Dec N→C) whilst the N-C direction of collagen runs from bottom to top (Col N-C↑), or in the opposite direction, Dec C→N as rendered in Figures 1–3. The logic of this approach was further confirmed by applying Zdock [43] to the decoron monomer and dimer with a single collagen microfibril. Despite the fact that a single microfibril lacks the proper context and model stringency, amongst the highest ranked docking alternatives from this approach were those two binding configurations that the fibril surface allows (Dec N→C or Dec C→N). For consistency, these same docking orientations were reproduced for each “receptor-ligand” model, where the “receptor” was: a microfibril (or more specifically the part of monomer 4 found in the *d* and *e*_1_ bands, Figures 1 and 2); two side by side microfibrils (representing a minimal fibril surface model with only the decoron concave facing collagen molecules), the common fibril conformation, or the wide fibril conformation for both the *d* and *e*_1_ band sites (Figures 2 and 3). The “ligand” models for each receptor model were: a decoron monomer or decoron dimer, in the Dec N→C or Dec C→N orientation (Figures 1 to [Fig pone-0007028-g002] 3). The term “receptor” is used in this context in the main text.

Each decoron model was manually docked to within 5 Å of each collagen binding site on the different receptor surfaces. The geometry of the receptor site ensured that two collagen monomers faced the concave interior of decorin (except for the single microfibril models that lacked the second monomer contact). The fibril surface models had in addition to the concave binding monomers, two to four monomers in position to contact the N and C-terminal tips of the decoron arms. Each model was then allowed to energy-minimize for at least 25,000 steps or until no further minimization occurred (whichever came last) with the collagen monomers set as rigid bodies, fixed in place. Following this step, the minimization was allowed to proceed a further 10,000–20,000 steps whilst allowing all atoms within 10 Å of the decoron molecule/s to relax. This was performed with the default options of the NAMD [44] extension of VMD [45].

### Free energy of association calculation

The relative strength of binding of each model was assessed with the “fastcontacts” server [34]. This provided a calculated output of the combined ‘free energy’ of the (4r) electrostatic interactions and energy of desolvation of each ligand-receptor complex (also referred to here and in the main text as the ‘energy of association’). The results of the server include a ranking of the top 20 strongest contributing ligand-receptor amino acid pairs to the free energy, and these are presented for the Dec N→C fibril binding models in Table S1

### H-bond detection

In addition to the electrostatic calculations, the location of the most persistent H-bonds were calculated for each model using the ‘WHATIF’ server [35]. The results are presented in Table S1 and Figure 4.

### Solvent-Accessible Surfaces and Electrostatic Calculations

The solvent accessible area and relative charge were both calculated using ‘spock’ with the default options except the surface polygon parameter which was set to 120 for improved surface definition and contrast [46]. The colouration scheme is per common convention: acidic red, basic blue.

### Amino acid sequences of collagen binding sites

The proposed decoron binding collagen sequence on the α1(I) chain 869-GAKGDRGETGP-879 (mature collagen sequence and numbering as found in RCSB coordinates 1Y0F/3HR2) [24], [33], [47] is found on the collagen molecule at ∼3.74D (*e*_1_ band). Similar sequences have been proposed to bind decoron, located at 3.87D, 2.74D and 2.65D respectively. We concentrated on those binding sites that we could see from our fibril model were located at the fibril surface in the mature stable form of the collagen fibril, in each D-period at ∼0.74D and ∼0.87D within the *e*_1_ and *d* microscopy bands respectively, the *d* band sequence being: 899-GKNGDRGEPGP-909. Figures 1 and 2 shows both of these sites to be located directly at the fibril surface, whilst the collagen monomer 3 sites (2.74D and 2.65D) are not so clearly exposed. The two non-binding sites chosen as controls were located 0.065D ∼4 nm above and below the *e*_1_ and *d* banding binding sites. A 3 amino acid sequence to sequence number misalignment in RCSB 1Y0F in the vicinity of position 876 of the α2 chain (part of the α2 sequence was moved 3 sequence positions towards the N-terminus) was corrected by threading the correct sequence into the correct sequence number positions at the d band site before the molecular modelling calculations described above were conducted. The *e*_1_ band and α1 sequences of both band sites were found to be correct and required no correction. The sequences were corrected for the coordinates of 1Y0F and uploaded as a corrected entry to the RCSB database under code 3HR2 (and the related entry 1YGV similarly updated to 3HQV).

The bovine and rat decoron binding sequences from collagen type I are shown in Table S2.

## Supporting Information

Figure S1 Number of hydrogen bonds in H-bonding network at decoron-collagen interface versus energy of association. The total includes intra as well as inter-molecular H-bonds. The orientation of the symbols indicates the monomeric versus dimer and Dec N→C versus Dec C→N orientations of the bound ligand (see key). Different colours represent the different receptor models: Black - single microfibril, Gray - two microfibrils, Blue - fibril surface wide conformation, Red - fibril surface common conformation.
(0.04 MB PDF)

Figure S2 A) Rendering of decoron molecule bound to the fibril surface (e_1_-band site) in the Dec C→N orientation; the four monomers in closest association with the docked decoron monomer are surface rendered in red, the decoron molecule is surface rendered in blue, the remaining collagen monomers are not shown. B) As A, except: worm traces through the peptide backbone are used to display the molecules instead of surface rendering and the decoron model is based on the ribonuclease inhibitor structure rather than the decoron crystal structure. Note the substantial molecular overlap that occurs when the decoron is docked to an individual collagen molecule, with the neighboring collagen molecules at the fibril surface. C) As B, except: the ribonuclease inhibitor based decoron molecule has been placed to avoid steric clashes, note that the receptor-ligand interface appears substantially less engaged than that seen in A.
(0.31 MB PDF)

Table S1 Principal decoron-collagen interactions. Decoron to collagen amino acid interactions are shown on the left, the matching positions (and colored highlights) on the right half of the table correspond to the calculated energy of association. Highlighted residues correspond to those that rank amongst the top 20 electrostatic pairs within one of the four conformations displayed (common and wide for the d and e_1_ band binding sites). Although the non-highlighted residues were estimated to contribute no more than −4.5 kJ/mol (which includes the desolvation cost), they were nevertheless estimated to form persistent H-bonds and cumulatively contribute to ligand-receptor stability.
(0.64 MB PDF)

Table S2 Differences between bovine and 1Y0F amino acid sequences at the decoron binding collagen sequences proposed previous to this study (note, monomer 1 is not involved in the interaction at the fibril surface). The decoron-collagen complex appears to form at the N-terminal end of each sequence (e.g. little to no engagement with the GPAG sequence at end of monomer 4 d or e_1_ band sequences).
(0.03 MB DOC)
