# Supplementary material for: Decorin Core Protein (Decoron) Shape Complements Collagen Fibril Surface Structure and Mediates Its Binding
Source: PLoS One. 2009 Sep 15;4(9):e7028. doi: 10.1371/journal.pone.0007028 (PMC2737631; doi:10.1371/journal.pone.0007028)
Supplement: Figure S1 — Number of hydrogen bonds in H-bonding network at decoron-collagen interface versus energy of association. The total includes intra as well as inter-molecular H-bonds. The orientation of the symbols indicates the monomeric versus dimer and Dec N→C versus Dec C→N orientations of the bound ligand (see key). Different colours represent the different receptor models: Black - single microfibril, Gray - two microfibrils, Blue - fibril surface wide conformation, Red - fibril surface common conformation. (0.04 MB PDF) [file pone.0007028.s001.pdf]

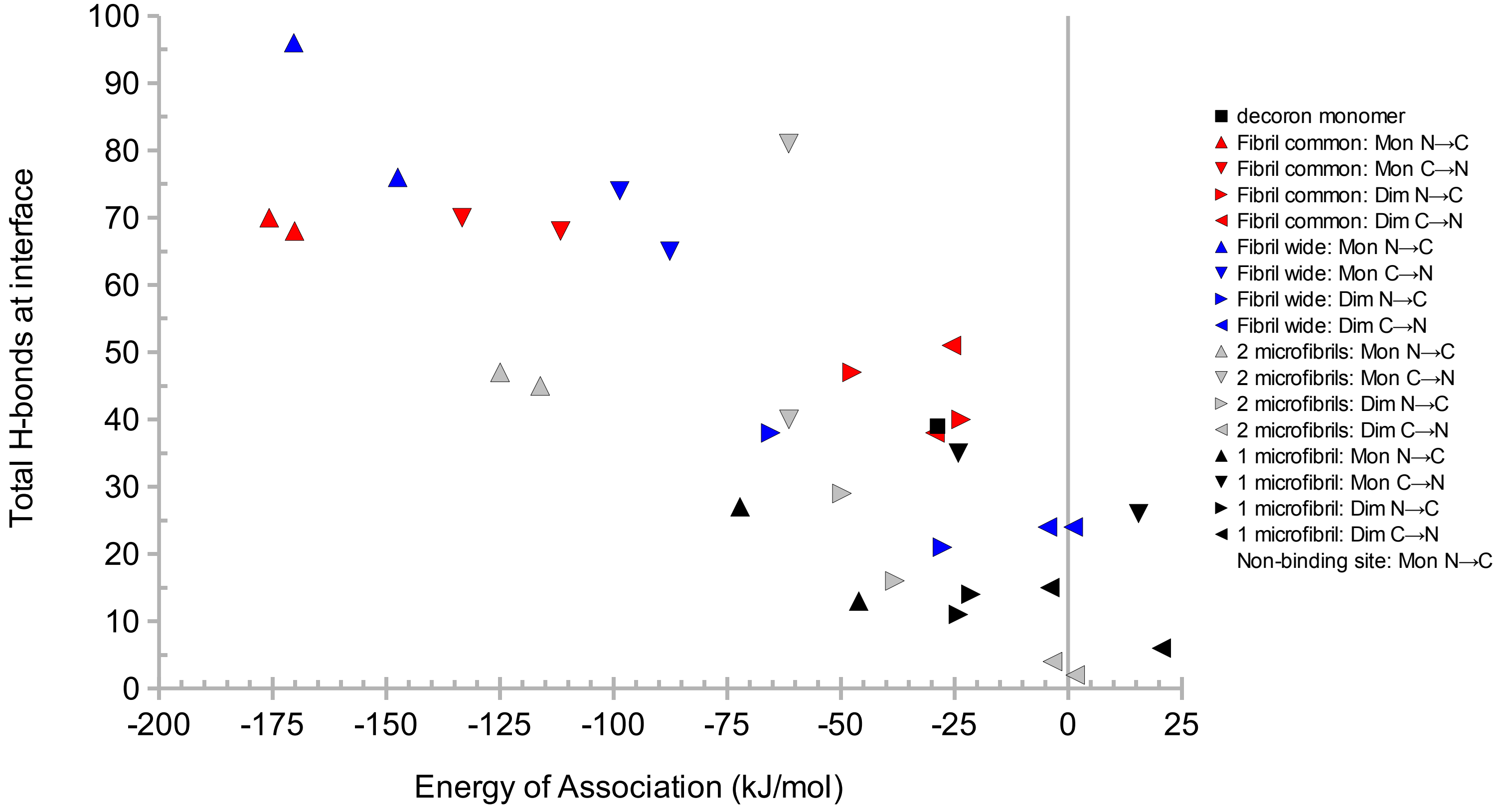

Figure S1

Number of hydrogen bonds in H-bonding network at decoron-collagen interface versus energy of association. The total includes intra as well as inter-molecular H-bonds. The orientation of the symbols indicates the monomeric versus dimer and Dec N→C versus Dec C→N orientations of the bound ligand (see key). Different colors represent the different receptor models:

Black – single microfibril, Gray – two microfibrils, Blue – fibril surface wide conformation, Red – fibril surface common conformation.
