# Supplementary material for: Decorin Core Protein (Decoron) Shape Complements Collagen Fibril Surface Structure and Mediates Its Binding
Source: PLoS One. 2009 Sep 15;4(9):e7028. doi: 10.1371/journal.pone.0007028 (PMC2737631; doi:10.1371/journal.pone.0007028)
Supplement: Figure S2 — A) Rendering of decoron molecule bound to the fibril surface (e1-band site) in the Dec C→N orientation; the four monomers in closest association with the docked decoron monomer are surface rendered in red, the decoron molecule is surface rendered in blue, the remaining collagen monomers are not shown. B) As A, except: worm traces through the peptide backbone are used to display the molecules instead of surface rendering and the decoron model is based on the ribonuclease inhibitor structure rather than the decoron crystal structure. Note the substantial molecular overlap that occurs when the decoron is docked to an individual collagen molecule, with the neighboring collagen molecules at the fibril surface. C) As B, except: the ribonuclease inhibitor based decoron molecule has been placed to avoid steric clashes, note that the receptor-ligand interface appears substantially less engaged than that seen in A. (0.31 MB PDF) [file pone.0007028.s002.pdf]

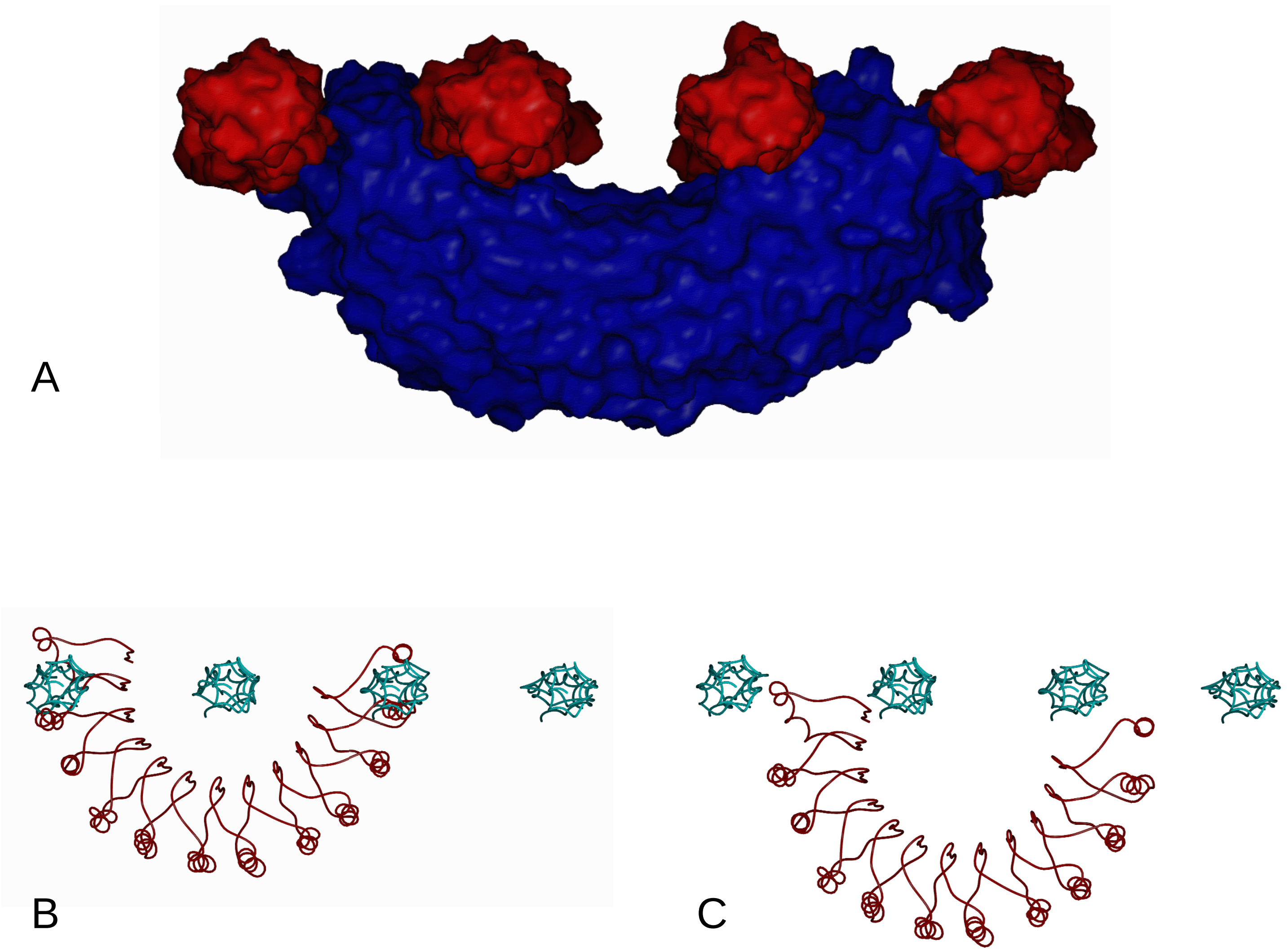

Figure S2

A) Rendering of decoron molecule bound to the fibril surface (e_1-band site) in the Dec C$\rightarrow$N orientation; the four monomers in closest association with the docked decoron monomer are surface rendered in red, the decoron molecule is surface rendered in blue, the remaining collagen monomers are not shown.

B) As A, except: worm traces through the peptide backbone are used to display the molecules instead of surface rendering and the decoron model is based on the ribonuclease inhibitor structure rather than the decoron crystal structure. Note the substantial molecular overlap that occurs when the decoron is docked to an individual collagen molecule, with the neighboring collagen molecules at the fibril surface.

C) As B, except: the ribonuclease inhibitor based decoron molecule has been placed to avoid steric clashes, note that the receptor-ligand interface appears substantially less engaged than that seen in A.
