# Supplementary material for: Decorin Core Protein (Decoron) Shape Complements Collagen Fibril Surface Structure and Mediates Its Binding
Source: PLoS One. 2009 Sep 15;4(9):e7028. doi: 10.1371/journal.pone.0007028 (PMC2737631; doi:10.1371/journal.pone.0007028)
Supplement: Table S1 — Principal decoron-collagen interactions. Decoron to collagen amino acid interactions are shown on the left, the matching positions (and colored highlights) on the right half of the table correspond to the calculated energy of association. Highlighted residues correspond to those that rank amongst the top 20 electrostatic pairs within one of the four conformations displayed (common and wide for the d and e1 band binding sites). Although the non-highlighted residues were estimated to contribute no more than −4.5 kJ/mol (which includes the desolvation cost), they were nevertheless estimated to form persistent H-bonds and cumulatively contribute to ligand-receptor stability. (0.64 MB PDF) [file pone.0007028.s003.pdf]

Decoron residues that bind collagen fibril surface			*d*-band: collagen residue (name, number, chain)					e_1-band: collagen residue (name, number, chain)						*d*-band: energy of association				e_1-band: energy of association				Decoron residues that bind collagen fibril surface		
LRR	residue	number	common		wide			common			wide			common		wide		common		wide		residue	number	LRR
N-term	ILE	6	K900A					Q860B						-12.56								ILE	6	N-term
	GLY	7	K900C	G665A										-8.41								GLY	7	
	GLU	9	K890B		K890B	N893B					R634A	R627B				-36.96				-29.73	-26.30	GLU	9	
	GLU	10			L891B	K890B	G892B										-34.96					GLU	10	
	GLU	14	L891B		G899A	K900C		A870A	K871A		A870A	K871A	H863B			-9.12		-9.44	19.84	-10.12		GLU	14	
	GLU	17	R894B											-16.38								GLU	17	
	ILE	18	R893B																			ILE	18	
	GLU	19	R894B	K900A	R894B			H863B	K864B					-6.25	-12.98	-7.22			-21.23			GLU	19	
	PRO	20			R894B																	PRO	20	
	MET	21	R894B		R894B						K871A					-10.66						MET	21	
	GLY	22									K871A											GLY	22	
	PRO	23	R894B								R874A											PRO	23	
	VAL	24	G902C	R894B	R894B			D873A	R874A						-8.40				8.00			VAL	24	
	CYS	25	D903C	R660B																		CYS	25	
	PRO	26						T869B														PRO	26	
	ARG	28	R904A								E642A			-8.38								ARG	28	
	HIS	29			R904C																	HIS	29	
	GLN	30	G902C	R904A	G661B	R904C		R402C			R402C					-21.65		-12.31				GLN	30	
	CYS	31	R894B		E659B	R894B								-12.62								CYS	31	
	HIS	32	R894B		K664A			K871C						-11.10		-12.23		-4.71				HIS	32	
1	LEU	33	R894B					K871C						-6.33				-15.67				LEU	33	1
	VAL	35									R402C											VAL	35	
	GLN	37	G905A	R904A				R867B			R402C							-16.64		-10.23		GLN	37	
	SER	39						E876C														SER	39	
	LYS	48			D903C											-14.62						LYS	48	
2	ASP	58						R867B										-5.89				ASP	58	2
	GLN	60			T907C	G905C		E876C			E876A							-13.30				GLN	60	
	ASN	61			T907C			E876C														ASN	61	
3	HIS	79			A898C			K864B										-14.84				HIS	79	3
	THR	80			D903C						K864B									-13.22		THR	80	
4	GLU	103						K871A			K871C	K864B						-23.09		-19.69	-17.65	GLU	103	4
	ARG	104			D903C											-19.04						ARG	104	
	TYR	106			K900C																	TYR	106	
	SER	108			E906C						E876A											SER	108	
	LYS	109	P909A		E906C						G878A					-43.66						LYS	109	
5	GLU	125			K900C											-44.16						GLU	125	5
	ARG	127									E866B									-41.67		ARG	127	
	HIS	129			R904C											-7.31						HIS	129	
	GLU	130			R904C						G878A					-19.04				-8.86		GLU	130	
7	ARG	177									E876A									-24.55		ARG	177	7
8	GLU	196			K900A			K864B								-28.31		-19.80				GLU	196	8
	HIS	198			K900C																	HIS	198	
	ASP	200									R867B									-11.17		ASP	200	
9	LYS	220	G905A	D903A		D903C					E866B				-7.75					-30.22		LYS	220	9
10	ARG	243	D903A		G899C			D873C						-24.31				5.20				ARG	243	10
	GLU	244	K900C		K900A			R867B	K871A		K871C			-33.56				-31.59	-7.27	-42.89		GLU	244	
	HIS	246									R867B											HIS	246	
	ASN	248						E876C			G871B	P870B						-10.31				ASN	248	
	ASN	249						E876C			G871B											ASN	249	
11	GLN	266	K900C		R894B			K871A			K871C	G872C				-7.72		-9.10		-19.15		GLN	266	11
	TYR	269	D903C		D903C																	TYR	269	
	ASN	272	A910A								N872B											ASN	272	
	TRY	288									K864B	G865B										TRY	288	
	SER	294	R894B																			SER	294	
12	TYR	295	R894B											-17.64								TYR	295	12
	SER	296	R894B	G902C	R894B			E866B			R874C	D873B		-7.42		-13.69				-10.43		SER	296	
	TYR	307	V903B		G908A	A902B		E876C	A880A		A880A	A406C										TYR	307	
	SER	313						E866B			R874A											SER	313	
	ARG	316									K864B									-12.67		ARG	316	
	CYS	317									K864B									-10.71		CYS	317	
	TYR	319									K864B									-13.63		TYR	319	
	VAL	320	A898C					K864B														VAL	320	
	ARG	321	D903A		D903A			D873A	E866B	K864B	D873A	D393B		-34.50		-32.67				-24.01		ARG	321	
C-term	ALA	323	R894B											-11.82								ALA	323	C-term
	VAL	324			R660B			K864B								-12.38		-10.61				VAL	324	
	GLN	325	R894B	R660B	R660B						R634A			-11.20	-8.45	-8.25				-13.53		GLN	325	
	LEU	326	R894B					R874A						-9.17								LEU	326	
	GLY	327	D903C																			GLY	327	
	TYR	329	D903C		R904C			R874A	T869B													TYR	329	

Table S1

Principal decoron-collagen interactions. Decoron to collagen amino acid interactions are shown on the left, the matching positions (and colored highlights) on the right half of the table correspond to the calculated energy of association. Highlighted residues correspond to those that rank amongst the top 20 electrostatic pairs within one of the four conformations displayed (common and wide for the *d* and e_1 band binding sites). Although the non-highlighted residues were estimated to contribute no more than -4.5 kJ/mol (which includes the desolvation cost), they were nevertheless estimated to form persistent H-bonds and cumulatively contribute to ligand-receptor stability.
