# Supplementary material for: Decorin Core Protein (Decoron) Shape Complements Collagen Fibril Surface Structure and Mediates Its Binding
Source: PLoS One. 2009 Sep 15;4(9):e7028. doi: 10.1371/journal.pone.0007028 (PMC2737631; doi:10.1371/journal.pone.0007028)
Supplement: Table S2 — Differences between bovine and 1Y0F amino acid sequences at the decoron binding collagen sequences proposed previous to this study (note, monomer 1 is not involved in the interaction at the fibril surface). The decoron-collagen complex appears to form at the N-terminal end of each sequence (e.g. little to no engagement with the GPAG sequence at end of monomer 4 d or e1 band sequences). (0.03 MB DOC) [file pone.0007028.s004.doc]

Table S2:

Differences between bovine and 1Y0F amino acid sequences at the decoron binding collagen sequences proposed previous to this study (note, monomer 1 is not involved in the interaction at the fibril surface). The decoron-collagen complex appears to form at the N-terminal end of each sequence (e.g. little to no engagement with the GPAG sequence at end of monomer 4 d or e_1_ band sequences).

0.87D (d-band)

monomer 4

1Y0F α1 m4 at .87D 900-KNGDRGEPGPAG-911

bov α1 m4 at .87D KSGDRGEPGPAG

S

1Y0F α2 m4 at .87D KLGNRGEOGPAG

bov α2 m4 at .87D KHGNRGEOGPAG

H

monomer 3

1Y0F α1 m3 at .87D 666-EPGDTGVKGDAG-677

bov α1 m3 at .87D EPGDAGAKGDAG

A A

1Y0F α2 m3 at .87D ERGTKGPVGEQG

bov α2 m3 at .87D ERGTKGPKGENG

N

monomer 2

1Y0F α1 m2 at .87D 432-KAGERGVPGPPG-443

bov α1 m2 at .87D KAGERGVPGPPG

1Y0F α2 m2 at .87D KPGEKGNVGLAG

bov α2 m2 at .87D KAGEKGHAGLAG

A HA

0.74 D (e_1_-band)

monomer 4

1Y0F α1 m4 at .74D 870-AKGDRGETGPAG-881

bov α1 m4 at .74D AKGDRGETGPAG

1Y0F α2 m4 at .74D HKGERGYPGNIG

bov α2 m4 at .74D HKGERGYPGNAG

A

monomer 3

1Y0F α1 m3 at .74D 636-APGDRGEAGPPG-647

bov α1 m3 at .74D APGDRGEPGPPG

P

1Y0F α2 m3 at .74D 629-IRGERGEPGPVG-640

bov α2 m3 at .74D SPGERGEVGPAG

S V A

monomer 2

1Y0F α1 m2 at .74D 402-RPGPAGPPGARG-413

bov α1 m2 at .74D RPGPPGPPGARG

P

1Y0F α2 m2 at .74D RPGPIGPAGPRG

bov α2 m2 at .74D RPGPIGPAGARG

A
